# Personality and Peripartum Changes in Perceived Social Support: Findings From Two Prospective-Longitudinal Studies in (Expectant) Mothers and Fathers

**DOI:** 10.3389/fpsyt.2021.814152

**Published:** 2022-01-20

**Authors:** Eva Asselmann, Susan Garthus-Niegel, Julia Martini

**Affiliations:** ^1^Faculty of Health, HMU Health and Medical University, Potsdam, Germany; ^2^Faculty of Medicine, Medical School Hamburg, Institute for Systems Medicine (ISM), Hamburg, Germany; ^3^Faculty of Medicine, Institute and Policlinic of Occupational and Social Medicine, Technische Universität Dresden, Dresden, Germany; ^4^Department of Child Health and Development, Norwegian Institute of Public Health, Oslo, Norway; ^5^Faculty of Psychology, School of Science, Institute of Clinical Psychology and Psychotherapy, Technische Universität Dresden, Dresden, Germany; ^6^Faculty of Medicine, Department of Psychiatry and Psychotherapy, Carl Gustav Carus University Hospital, Technische Universität Dresden, Dresden, Germany

**Keywords:** pregnancy, social support, personality, relationship processes, longitudinal, MARI study, DREAM study

## Abstract

**Objective:**

The aim of this study was to examine changes in perceived social support from early pregnancy to 2 years postpartum and to test whether these changes (a) differ between mothers and fathers or (b) vary as a function of the Big Five personality traits.

**Background:**

Higher peripartum social support in (expectant) mothers and fathers has been associated with fewer complications during pregnancy and delivery as well as better parental and offspring health.

**Methods:**

Prospective-longitudinal data from two regional-epidemiological samples from Germany were used: MARI (*N* = 396, including *n* = 293 mothers and *n* = 103 fathers) and DREAM (*N* = 2,819, including *n* = 1,689 mothers and *n* = 1,130 fathers). The Big Five personality traits were assessed during pregnancy in MARI as well as 8 weeks after the anticipated birth date in DREAM with short forms of the Big Five Inventory. Perceived social support was assessed during pregnancy, 4 months postpartum, and 16 months postpartum in MARI as well as during pregnancy, 14 months postpartum, and 2 years postpartum in DREAM using the short version of the Social Support Questionnaire.

**Results:**

Multilevel analyses revealed that perceived social support decreased across the peripartum period, and this decrease did not differ between mothers and fathers. More extraverted, emotionally stable, agreeable, conscientious, and open parents perceived higher levels of social support across the peripartum period. The peripartum decrease of perceived social support was smaller in parents who were more extraverted.

**Conclusion:**

Our findings suggest that especially extraversion plays an important role for high and stable levels of perceived social support across the peripartum period.

**Implications:**

Particularly highly introverted parents might profit from targeted social support interventions.

## Introduction

Before and after the birth of a child, (expectant) mothers and fathers are faced with far-reaching life changes and new responsibilities (1–4). Social support from significant others (e.g., partners, parents, siblings, friends, colleagues, or health care professionals) during this challenging time plays an important role not only for their own well-being and functioning but also for the development of their offspring: Higher perceived social support during the peripartum period has been associated with better parental physical and mental health (5–7), fewer complications during pregnancy and delivery (8, 9), as well as more favorable developmental and health outcomes in the offspring (10). Conversely, lower social support has been linked to increased stress, anxiety, and depressive symptoms during pregnancy and after delivery (5, 7, 11, 12). Previous evidence even suggests that a lack of social support is one of the strongest environmental risk factors of postpartum depression (13–15).

Therefore, a major research task is to examine what contributes to high and stable levels of perceived social support across the peripartum period. Such research provides important cues for an early identification of high-risk groups of (expectant) parents as well as early targeted interventions to foster peripartum social support.

### Peripartum Changes in Perceived Social Support

After the birth of a child, the social network size of new parents tends to decrease (16), while the intensity of support from close relatives (e.g., partner or parents) tends to increase (17–19). Nonetheless, the gap between desired and actual support might widen after delivery due to new childcare responsibilities and parenting stress (and in turn less time to meet friends) (6). Consistent with this assumption, prospective-longitudinal studies found that perceived social support decreased across the transition to parenthood (6, 18, 20). However, most of these studies focused on mothers, and little is known about peripartum changes in perceived social support in fathers.

### Gender Differences With Respect to Peripartum Changes in Perceived Social Support

In line with traditional gender stereotypes (21), men might engage less in childcare than women. Therefore, fathers might experience less parenting stress and thus a smaller gap between desired and actual levels of support after childbirth than mothers. On the other hand, men still frequently function as the breadwinner of the family. Therefore, one could also speculate whether fathers feel less familiar with childcare and family issues, are more overwhelmed by their dual role, and thus perceive a greater lack of support after childbirth than mothers (22). Taken together, it is both plausible to assume that fathers experience a smaller or larger decrease of perceived social support after childbirth than mothers. Additional research is needed to resolve this question.

### The Role of Personality for Changes in Perceived Social Support Across the Peripartum Period

Furthermore, levels and changes of perceived social support across the peripartum period might vary as a function of parents' personality. Personality refers to individual differences in thoughts, feelings, and behaviors (23) that can be described by the Big Five personality traits extraversion, emotional stability, agreeableness, conscientiousness, and openness to experience (24).

Previous research suggests that higher extraversion, emotional stability, agreeableness, conscientiousness, and openness relate to higher perceived social support (25). Extraverted individuals tend to be cheerful, energetic, outgoing, and socially active and experience more positive affect (25, 26). Thus, more extraverted mothers and fathers might handle challenging situations in family life more easily, need less social support, and experience their social support as more satisfactory (27). At the same time, more extraverted parents might have larger social networks (e.g., more friends) and better access to potential sources of support (5). Finally, they might be more likely to actively communicate their needs and directly seek for help, leading to higher levels of actual support in times of trouble.

Emotionally stable individuals tend to be resistant against stress and experience less negative affect (25, 26). Therefore, more emotionally stable mothers and fathers might have fewer difficulties to adjust to the new family situation (5, 28–30). They might cope with hassles due to a lack of sleep and lower autonomy more easily, require less help from others, and perceive their social support as less deficient.

Agreeable individuals are compliant, kind, and supportive (25). Because these characteristics tend to be beneficial in close relationships (27), more agreeable mothers and fathers might have stronger ties to their partner, close relatives, and friends and receive more social support during the peripartum period (31).

Conscientious individuals are careful, competent, diligent, and organized. Moreover, higher conscientiousness relates to higher mastery and more effective self-regulation (26, 32). Thus, more conscientious mothers and fathers might re-structure their daily routines and manage their new responsibilities more effectively and need less support. At the same time, more conscientious individuals might act more reliably toward others, which might foster secure relationships and reliable support.

Individuals who score high on openness tend to be open-minded, adventurous, and creative (33, 34). Therefore, more open mothers and fathers might “embrace” their new role more, find unconventional ways to cope with stress, and need less support (35). In addition, their social networks might be more diverse, providing different sources of support (e.g., instrumental and emotional) for different problems.

Taken together, prior findings suggest that (a) perceived social support tends to decrease from pregnancy to postpartum and that (b) higher extraversion, emotional stability, agreeableness, conscientiousness, and openness relate to higher levels of perceived support. However, whether these personality characteristics relate to more favorable changes in perceived social support across the peripartum period (i.e., a greater increase or smaller decrease of support) remains an open question so far.

### Aims of the Study

We used data from two prospective-longitudinal studies to examine changes in perceived social support from early pregnancy to 2 years postpartum and to test whether these changes (a) differ between mothers and fathers or (b) vary as a function of the Big Five personality traits. We hypothesized that social support decreases from pregnancy to postpartum and that this decrease is smaller in more extraverted, emotionally stable, agreeable, conscientious, and open parents. Gender differences between mothers and fathers were tested exploratorily. The findings from both studies were compared to evaluate the replicability, robustness, and reproducibility of the effects (36).

## Methods

Prospective-longitudinal data from two regional-epidemiological samples (MARI and DREAM) were combined.

### MARI

The Maternal Anxiety in Relation to Infant Development (MARI) Study is a prospective-longitudinal regional-epidemiological study in (expectant) mothers and their partners from the area of Dresden, Germany (01/2009–09/2012). The study objective was to investigate the role of anxiety and depressive disorders prior to, during, and after pregnancy for perinatal outcomes, maternal health, and offspring development. Seven assessment waves with standardized diagnostic interviews, questionnaires, and observations were conducted: T1 (baseline, week 10–12 of gestation), T2 (week 22–24 of gestation), T3 (week 35–37 of gestation), T4 (10 days postpartum), T5 (2 months postpartum), T6 (4 months postpartum), and T7 (16 months postpartum). More detailed information on the aims, methods, design, and inclusion/ exclusion criteria of the MARI study, including a detailed study flow chart, has been presented elsewhere (37–39).

Informed consent was obtained from all participants. The study was approved by the Ethics Committee of the Faculty of Medicine of the Technische Universität Dresden (No: EK 94042007) and carried out in accordance with the Helsinki Declaration of 1975, as revised in 2008 and 2013.

#### Sample of (Expectant) Mothers

Overall, 533 expectant mothers were sampled from 22 gynecological outpatient settings in and around Dresden, Germany, and screened for inclusion and exclusion criteria. Fifty women were not enrolled due to the following exclusion criteria: Gestational age > 12 weeks (*N* = 8); age < 18 or > 40 years (*N* = 8); multiple pregnancy (*N* = 2); history of more than three spontaneous abortions, (induced) termination of the pregnancy, still birth, or infant impairment (*N* = 2); invasive fertility treatment (*N* = 9); severe physical disease, microsomia, or skeletal malformation (*N* = 6); substance abuse or heroin substitution during the past 6 months (*N* = 0); severe psychiatric illness (*N* = 2); expectation to leave the area of Dresden (*N* = 6); insufficient German skills (*N* = 7). Nine women did not participate due to spontaneous abortions before T1, 10 because their partner did not agree, 154 due to a lack of time, and four due to unknown reasons.

Finally, 306 expectant mothers were enrolled in the study, and 274 were retained until T7 (retention rate: 89.5 %). The participation of eight women ended after T1 due to a spontaneous abortion or termination of the pregnancy. During the study, three women moved away from the area of Dresden, five women could not be reached anymore by phone, postal, or personal contact, nine women reported a lack of time or interest in further participation, and seven women refused to be contacted again for further follow-up assessments. Some retained women did not participate in single assessments, for example, due to a preterm delivery, sickness, or a lack of time. Detailed information on sociodemographic, gynecological, and clinical characteristics of the sample of mothers has been previously published (38, 39).

#### Sample of (Expectant) Fathers

From week 22 to 24 of gestation, all 306 expectant mothers were asked whether the study personnel may approach their partner to participate in the study, and 134 (43.8 %) indicated that their partner may be contacted by phone. Of these partners, 109 met the inclusion criteria (sufficient German skills as well as willingness and time to participate) and agreed to participate. All 109 partners were men. In total, 109 (expectant) fathers participated at T2, 96 at T5, 91 at T6, and 26 at T7.

(Expectant) mothers with (*N* = 109) and without (*N* = 197) a participating partner differed in terms of sociodemographic, psychosocial, and neonatal characteristics (37). Specifically, expectant mothers with (vs. without) a participating partner were younger, better educated, and more often primiparous. Moreover, women with (vs. without) a participating partner reported higher postpartum levels of social support and a better partnership quality (37).

### DREAM

The Dresden Study on Parenting, Work, and Mental Health (“**DR**esdner Studie zu **E**lternschaft, **A**rbeit und **M**entaler Gesundheit,” **DREAM**) is a prospective-longitudinal cohort study in (expectant) mothers and their partners, recruited from June 2017 to the end of 2020 from the area of Dresden, Germany (06/2017–ongoing). The study objective was to prospectively examine the association between parental work participation, role distribution, and stress across the peripartum period, including their effects on perinatal outcomes and family health. So far, six questionnaire-based assessment waves are being conducted: T1 (baseline, during pregnancy, finished), T2 (8 weeks after the anticipated birth date), T3 (14 months postpartum), T4 (2 years postpartum), T5 (3 years postpartum), and T6 (4.5 years postpartum, starting in the end of 2021). More detailed information on the aims, methods, design, and inclusion/ exclusion criteria of the DREAM study, including a detailed study flow chart, has been published elsewhere (40).

Informed consent was obtained from all participants. The study was approved by the Ethics Committee of the Faculty of Medicine of the Technische Universität Dresden (No: EK 278062015) and carried out in accordance with the Helsinki Declaration of 1975, as revised in 2008 and 2013. The data were collected and are managed using Research Electronic Data Capture (REDCap), hosted at the “Koordinierungszentrum für Klinische Studien” at the Faculty of Medicine of the Technische Universität Dresden (41, 42).

#### Sample

Expectant mothers and their partners were recruited during pregnancy, predominately at information sessions in hospitals and birth preparation courses in and around Dresden, Germany. Inclusion criteria were a current pregnancy, being a resident in the Dresden area, and sufficient German skills to complete the study questionnaires. Specific sub-groups (e.g., women with multiple pregnancies and same-sex couples) were not excluded to obtain a diverse sample and maximize the generalizability to the general population of expectant parents. The data collection of the DREAM study is currently still ongoing except for T1. Thus, the sample sizes from T2 onwards do not reflect the finally retained participants but only refer to those parents who have been due at the time of the data extraction. The current study is based on version 8 of the quality-assured data files, released for research in March 2021. Based on this version, 2,209, 1,967, 1,428, and 797 mothers as well as 1,608, 1,343, 969, and 544 partners participated at T1, T2, T3, and T4, respectively. Detailed information on sociodemographic, gynecological, and clinical sample characteristics has been presented elsewhere (40).

#### Assessment of Personality

In MARI, the Big Five were assessed at T2 (week 22–24 of gestation) with the German version of the BFI-K, the 21-item short form of the Big Five Inventory (43). The BFI-K contains five items for openness and four items for each of the other traits, labeled from 1 = “strongly disagree” to 5 = “strongly agree.”

In DREAM, the Big Five were assessed at T2 (8 weeks after the anticipated birth date) with the German version of the BFI-S, the 15-item short form of the Big Five Inventory (John et al., 1991) (44, 45). The BFI-S contains three items per trait, labeled from 1 = “strongly disagree” to 7 = “strongly agree.” The reliability and validity of the BFI-K (43) and BFI-S (46–49) have been supported previously. Both measures are well established and frequently used for the assessment of the Big Five.

#### Assessment of Perceived Social Support

Perceived social support was assessed in MARI at T2 (in week 22–24 of gestation), T6 (4 months postpartum), and T7 (16 months postpartum) and in DREAM at T1 (during pregnancy), T3 (14 months postpartum), and T4 (2 years postpartum) with the short version (K-14) of the Social Support Questionnaire (F-SozU) Form A (50). The F-SozU Form A K-14 consists of 14 items (labeled 1 = “does not apply,” 2 = “does rather not apply,” 3 = “does partially apply,” 4 = “does apply,” and 5 = “does exactly apply”). These items refer to instrumental and emotional support, social integration, satisfaction with social support, and availability of confidants. The total score indicates participants' total levels of perceived social support. The internal consistency, test-retest reliability, as well as convergent and discriminant validity of the F-SozU have been shown to be good (50).

### Statistical Analyses

Only individuals with information on personality (at T2 in MARI and DREAM) and perceived social support at any wave (at T2, T6, and/ or T7 in MARI and at T1, T3, and/ or T4 in DREAM) were considered. In MARI, this resulted in a total sample of 396 individuals (*N* = 293 mothers and *N* = 103 fathers). Specifically, 396 individuals (*N* = 293 mothers and *N* = 103 fathers) provided information on perceived social support at T2, 358 (*N* = 281 mothers and *N* = 77 fathers) at T6, and 280 (*N* = 255 mothers and *N* = 25 fathers) at T7.

In DREAM, this resulted in a sample of 3,172 individuals (*N* = 1,897 mothers and *N* = 1,275 partners). Of these individuals, 335 were excluded because they did not complete T1 during (but after) pregnancy. In addition, 9 partners were excluded because they were female and 7 partners were excluded because they were not the biological father of the child. Finally, 14 individuals were excluded due to missing information on their date of birth. Thus, the final sample in DREAM consisted of 2,819 individuals (*N* = 1,689 mothers and *N* = 1,130 fathers). Specifically, 2,819 individuals (*N* = 1,689 mothers and *N* = 1,130 fathers) provided information on perceived social support at T1, 1,829 (*N* = 1,122 mothers and *N* = 707 fathers) at T3, and 914 (*N* = 556 mothers and *N* = 358 fathers) at T4.

The analyses were performed with Stata 14 (51). Multilevel analyses with measurement occasions (Level 1) nested within persons (Level 2) nested within couples (Level 3) were used. Specifically, we simultaneously regressed the perceived social support score (standardized across all waves) on age, gender, an early-postpartum variable, a late-postpartum variable, and two interaction terms (gender × early-postpartum and gender × late-postpartum). The early-postpartum variable was used to test for changes in perceived social support from pregnancy to early postpartum (i.e., 4 months postpartum in MARI and 14 months postpartum in DREAM). The late-postpartum variable was used to test for further changes in perceived social support until late postpartum (i.e., 16 months postpartum in MARI and 2 years postpartum in DREAM). The interaction terms (gender × early/late-postpartum) were included to test whether these short- and long-term changes differed between mothers and fathers.

To test whether levels and changes in perceived social support varied by personality, we built the same model and additionally included (a) the respective Big Five trait as well as interaction terms of the respective Big Five trait with (b) gender, (c) the early-postpartum variable, and (d) the late-postpartum variable. A separate model was built for each Big Five trait to avoid multicollinearity. The interaction term with gender was used to test whether the role of the respective Big Five trait for perceived social support differed between mothers and fathers. The interaction terms with the early- and late-postpartum variable, respectively, were used to test whether the short- and/ or long-term changes in perceived social support varied by the respective Big Five trait. Table 1 summarizes how each predictor was defined and coded. The alpha level was set at.05. Because each analysis refers to another research question, we did not control for multiple testing (52).

**Table 1:** Description and coding of the predictors.

**Predictor**	**Used to examine…**	**Coding**
Age (Level 2 predictor)	Linear age effects	• Age in years • Centered across all waves
Gender (Level 2 predictor)	Differences in perceived social support between mothers and fathers	• Coded with 0 in mothers and 1 in fathers • Centered across all waves
Early-postpartum (Level 1 predictor)	Changes in perceived social support from pregnancy to early postpartum	• Coded with 1 at T6 (4 months postpartum) in MARI and T3 (14 months postpartum) in DREAM • Coded with 0 at all other waves
Late-postpartum (Level 1 predictor)	Changes in perceived social support from pregnancy/early postpartum to late postpartum	• Coded with 1 at T7 (16 months postpartum) in MARI and T4 (2 years postpartum) in DREAM • Coded with 0 at all other waves
Big Five trait (Level 2 predictor)	Personality effects	• Respective personality trait score (extraversion, emotional stability, agreeableness, conscientiousness, or openness, respectively) • Standardized across all waves

*Multilevel analyses with measurement occasions (Level 1) nested within persons (Level 2) nested within couples (Level 3) were used*.

## Results

### Sample Characteristics

Baseline sample characteristics for mothers and fathers of the MARI and DREAM sample are shown in Tables 2, 3. In MARI, the correlation of perceived social support during pregnancy with perceived social support 4 months and 16 months postpartum was *r* = 0.75 and *r* = 0.68, respectively. The correlation of perceived social support 4 months and 16 months postpartum was *r* = 0.74. Perceived social support during pregnancy was slightly higher in parents who did (*M* = 4.39, *SD* = 0.52) vs. did not (*M* = 4.24, *SD* = 0.60) participate in the study from pregnancy until 16 months postpartum, *t*_(394)_ = −2.48, *p* = 0.013. That is, participants with lower initial levels of perceived social support were more likely to drop out.

**Table 2:** Baseline sample characteristics of the MARI sample (*N* = 396).

**Sample characteristic**	**(Expectant) mothers**		**(Expectant) fathers**	
	**(*****N*** **= 293)**		**(*****N*** **= 103)**	
Age (*M, SD*)	28.06	4.41	31.32	5.88
Education (*N, %*)				
No degree or 9th grade	20	6.83	7	6.80
10th grade	74	25.26	25	24.27
High school	106	36.18	31	30.10
University	93	31.74	40	38.83
Marital status (*N*, %)				
Married	106	36.18	41	39.81
Never married	177	60.41	58	56.31
Separated/ widowed/ divorced	10	3.41	4	3.88
Working time (*N*, %)				
Full-time	116	39.59	75	72.82
Part-time	80	27.30	5	4.85
Currently not working	97	33.11	23	22.33
Monthly household income after taxes (*N, %*)				
< 500 Euros	21	7.17	9	8.74
500–1,000 Euros	103	35.15	42	40.78
1,500–2,500 Euros	91	31.06	29	28.16
2,500–3,500 Euros	53	18.09	15	14.56
3,500–4,500 Euros	18	6.14	6	5.83
More than 4,500 Euros	7	2.39	2	1.94
Big Five traits at T2 (*M, SD*)				
Extraversion	3.60	0.83	3.31	0.87
Emotional stability	3.36	0.79	3.68	0.71
Agreeableness	3.35	0.67	3.31	0.72
Conscientiousness	3.83	0.58	3.75	0.57
Openness	3.78	0.63	3.74	0.75

*M, mean; SD, standard deviation*.

**Table 3:** Baseline sample characteristics of the DREAM sample (*N* = 2,819).

**Sample characteristic**	**(Expectant) mothers**		**(Expectant) fathers**	
	**(*****N*** **= 1,689)**		**(*****N*** **= 1,130)**	
Age (*M, SD*)	30.24	3.98	32.59	4.95
Education (*N*, %)				
No degree or 9th grade	15	0.89	39	3.45
10th grade	344	20.37	262	23.19
High school	365	21.61	210	18.58
University	953	56.42	606	53.63
Other/unknown/missing data	12	0.71	13	1.15
Marital status (*N*, %)				
Married	752	44.52	531	46.99
Unmarried	892	52.81	557	49.29
Widowed/divorced	41	2.43	38	3.36
Unknown/missing data	4	0.24	4	0.35
Working time (*N*, %)				
Full-time	767	45.41	956	84.6
Part-time	259	15.33	82	7.26
Irregular	40	2.37	30	2.65
School/ university/ training	64	3.79	35	3.1
Currently not working	472	27.95	13	1.15
Other/unknown/missing data	87	5.15	14	1.24
Monthly individual income after taxes (*N, %*)				
≤ 450 Euros	53	3.14	25	2.21
451–850 Euros	46	2.72	12	1.06
851–1,500 Euros	392	23.21	154	13.63
1,501–2,500 Euros	890	52.69	612	54.16
>2,500 Euros	216	12.79	281	24.87
Other/ unknown/ missing data	92	5.45	46	4.07
Big Five traits at T2 (*M, SD*)				
Extraversion	4.75	1.32	4.68	1.31
Emotional stability	4.23	1.23	4.92	1.14
Agreeableness	5.45	0.91	5.33	0.88
Conscientiousness	5.62	0.93	5.31	0.95
Openness	4.70	1.22	4.70	1.13

*M, mean; SD, standard deviation*.

In DREAM, the correlation of perceived social support during pregnancy with perceived social support 14 months and 2 years postpartum was *r* = 0.72 and *r* = 0.65, respectively. The correlation of perceived social support 14 months and 2 years postpartum was *r* = 0.72. Perceived social support during pregnancy did not differ significantly between parents who did (*M* = 4.30, *SD* = 0.59) and did not (*M* = 4.28, *SD* = 0.60) participate until 2 years postpartum, *t*_(2,817)_ = −0.56, *p* >0.05. That is, attrition did not vary by initial levels of perceived social support.

### Peripartum (Changes in) Perceived Social Support in Mothers and Fathers

Means and standard deviations for perceived social support at different assessment waves in the MARI and DREAM sample are shown in Table 4. In both samples, perceived social support was lower in fathers vs. mothers (MARI: β = −0.25, DREAM: β = −0.15; Table 5). Perceived social support decreased from pregnancy to early postpartum (i.e., 4 months postpartum in MARI: β = −0.09; 14 months postpartum in DREAM: β = −0.18), and decreased further until late postpartum (i.e., 16 months postpartum in MARI: β = −0.15; 2 years postpartum in DREAM: β = −0.19). The interactions with gender were not significant (all *p*-values > 0.05). That is, neither in MARI nor in DREAM, these changes differed between (expectant) mothers and fathers.

**Table 4:** Means and standard deviations of perceived social support at different assessment waves for (expectant) mothers and fathers in *MARI (N* = *396) and DREAM (N* = *2,819)*.

	**Perceived social support**											
	**MARI (*****N*** **= 396)**						**DREAM (*****N*** **= 2,819)**					
	**T2 (*****N*** **= 396)**		**T6 (*****N*** **= 358)**		**T7 (*****N*** **= 280)**		**T1 (*****N*** **= 2,819)**		**T3 (*****N*** **= 1,829)**		**T4 (*****N*** **= 914)**	
	**M**	**SD**	**M**	**SD**	**M**	**SD**	**M**	**SD**	**M**	**SD**	**M**	**SD**
Total sample	4.34	0.55	4.30	0.61	4.31	0.59	4.29	0.60	4.17	0.69	4.17	0.70
(Expectant) mothers	4.37	0.53	4.33	0.62	4.32	0.59	4.33	0.57	4.23	0.66	4.22	0.67
(Expectant) fathers	4.25	0.60	4.19	0.54	4.17	0.60	4.22	0.63	4.07	0.73	4.10	0.73

*M, mean; SD, standard deviation. In MARI, T2 was conducted in week 22–24 of gestation, T6 four months postpartum, and T7 16 months postpartum. In DREAM, T2 was conducted during pregnancy, T3 14 months postpartum, and T4 two years postpartum*.

**Table 5:** Peripartum (changes in) perceived social support, including differences between (expectant) mothers and fathers, in MARI (*N* = 396) and DREAM (*N* = 2,819).

	**Perceived social support**							
	**MARI (*****N*** **= 396)**				**DREAM (*****N*** **= 2,819)**			
**Predictor**	**β**	**95% CI**		**p**	**β**	**95% CI**		**p**
Gender (fathers vs. mothers)	**−0.25**	**−0.47**	**−0.03**	**0.025**	**−0.15**	**−0.21**	**−0.08**	**<0.001**
Early-postpartum	**−0.09**	**−0.17**	**−0.02**	**0.018**	**−0.18**	**−0.22**	**−0.15**	**<0.001**
Late-postpartum	**−0.15**	**−0.24**	**−0.06**	**0.001**	**−0.19**	**−0.24**	**−0.14**	**<0.001**
Gender × early-postpartum	−0.14	−0.32	0.05	0.145	−0.06	−0.14	0.01	0.071
Gender × late-postpartum	−0.12	−0.39	0.15	0.392	−0.04	−0.13	0.06	0.444

*β, beta-coefficient from multilevel analyses with measurement occasions (Level 1) nested within persons (Level 2) nested within couples (Level 3), adjusted for age at baseline. CI, confidence interval; p, p-value. Early-postpartum effect: Changes in perceived social support from pregnancy to early postpartum (i.e., 4 months postpartum in MARI and 14 months postpartum in DREAM). Late-postpartum effect: Further changes in perceived social support until late postpartum (i.e., 16 months postpartum in MARI and 2 years postpartum in DREAM). Significant effects (p < 0.05) are printed in bold*.

### The Role of Personality

Examining the role of personality revealed positive main effects for each of the Big Five personality traits in both studies (Table 6): Higher extraversion (MARI: β = 0.35, DREAM: β = 0.27), emotional stability (MARI: β = 0.30, DREAM: β = 0.19), agreeableness (MARI: β = 0.23, DREAM: β = 0.17), conscientiousness (MARI: β = 0.18, DREAM: β = 0.07), and openness (MARI: β = 0.10, DREAM: β = 0.12) were associated with higher levels of perceived social support across the peripartum period. The interactions with gender were not significant (all *p*-values >0.05). That is, neither in MARI nor in DREAM, these changes differed between mothers and fathers.

**Table 6:** Associations of the Big Five personality traits with peripartum (changes in) perceived social support in MARI (*N* = 396) and DREAM (*N* = 2,819).

	**Perceived social support**							
	**MARI (*****N*** **= 396)**				**DREAM (*****N*** **= 2,819)**			
**Predictor**	**β**	**95% CI**		**p**	**β**	**95% CI**		**p**
Extraversion (main effect)	**0.35**	**0.26**	**0.44**	**<0.001**	**0.27**	**0.24**	**0.30**	**<0.001**
Extraversion × gender	0.04	−0.14	0.23	0.643	0.03	−0.03	0.10	0.279
Extraversion × early-postpartum	0.03	−0.04	0.11	0.414	**0.04**	**0.01**	**0.08**	**0.011**
Extraversion × late-postpartum	0.05	−0.04	0.13	0.265	0.02	−0.03	0.06	0.515
Emotional stability (main effect)	**0.30**	**0.21**	**0.40**	**<0.001**	**0.19**	**0.15**	**0.22**	**<0.001**
Emotional stability × gender	0.14	−0.07	0.35	0.204	−0.01	−0.08	0.06	0.754
Emotional stability × early-postpartum	0.01	−0.07	0.09	0.829	0.01	−0.03	0.04	0.643
Emotional stability × late-postpartum	0.05	−0.03	0.13	0.253	−0.01	−0.06	0.04	0.748
Agreeableness (main effect)	**0.23**	**0.13**	**0.32**	**<0.001**	**0.17**	**0.14**	**0.21**	**<0.001**
Agreeableness × gender	−0.18	−0.37	0.01	0.064	0.04	−0.03	0.10	0.268
Agreeableness × early-postpartum	0.03	−0.04	0.11	0.383	−0.01	−0.04	0.03	0.745
Agreeableness × late-postpartum	0.00	−0.09	0.08	0.943	−0.03	−0.08	0.01	0.141
Conscientiousness (main effect)	**0.18**	**0.08**	**0.28**	**<0.001**	**0.07**	**0.03**	**0.11**	**<0.001**
Conscientiousness × gender	0.04	−0.16	0.25	0.680	−0.06	−0.12	0.01	0.088
Conscientiousness × early-postpartum	0.00	−0.08	0.08	0.988	0.01	−0.03	0.04	0.624
Conscientiousness × late-postpartum	−0.02	−0.11	0.06	0.611	0.01	−0.04	0.06	0.660
Openness (main effect)	**0.10**	**0.00**	**0.20**	**0.042**	**0.12**	**0.09**	**0.16**	**<0.001**
Openness × gender	0.16	−0.02	0.34	0.088	0.05	−0.01	0.12	0.114
Openness × early-postpartum	0.00	−0.08	0.07	0.980	0.02	−0.01	0.06	0.193
Openness × late-postpartum	−0.05	−0.13	0.04	0.278	−0.01	−0.06	0.03	0.533

*β, beta-coefficient from multilevel analyses with measurement occasions (Level 1) nested within persons (Level 2) nested within couples (Level 3), adjusted for age at baseline and all predictors of the main model in Table 2 (the main model was repeated five times, i.e., separately for each Big Five trait). CI, confidence interval; p, p-value. Early-postpartum effect: Changes in perceived social support from pregnancy to early postpartum (i.e., 4 months postpartum in MARI and 14 months postpartum in DREAM). Late-postpartum effect: Further changes in perceived social support until late postpartum (i.e., 16 months postpartum in MARI and 2 years postpartum in DREAM). Significant effects (p < 0.05) are printed in bold*.

In DREAM, there was a significant interactive effect between extraversion and the early-postpartum variable (β = 0.04), indicating that more extraverted parents experienced a smaller decrease of perceived social support from pregnancy to 14 months postpartum. However, no such interactive effect was found in MARI (*p*-value >0.05). Moreover, there was no evidence that any other peripartum changes in perceived social support varied as a function of the Big Five personality traits (all *p*-values >0.05).

## Discussion

These analyses based on data from two prospective-longitudinal studies aimed to examine (a) how perceived social support changes across the peripartum period in (expectant) mothers and fathers and (b) whether these changes vary by personality. Our main findings were that both mothers and fathers experienced a decrease of perceived social support from pregnancy to early and late postpartum. More extraverted, emotionally stable, agreeable, conscientious, and open individuals perceived higher levels of peripartum social support. In parents who scored higher on extraversion, the peripartum decrease of perceived social support was smaller.

First, we found that fathers experienced lower levels of perceived social support compared to mothers. As suggested by previous research (53), men might have fewer emotionally close relationships than women and thus fewer sources of support. In line with traditional gender stereotypes (21), fathers might also less often ask for help and be perceived as less vulnerable than mothers (54), which could lead to lower levels of actual social support. Compared to women, men still function more frequently as the breadwinner of the family. Therefore, they might on average feel less familiar with childcare and family issues and tend to be overwhelmed more by their dual role (22). Taken together, these potential reasons might explain why father experienced lower overall levels of perceived social support compared to mothers.

Second, our findings revealed that perceived social support decreased from pregnancy to early postpartum and from early to late postpartum. These results are consistent with previous findings (6, 18, 20) and might be explained by higher childcare and parenting responsibilities after delivery. These responsibilities and associated distress might accumulate and lead to a larger gap between desired and actual support over time (6). Our findings considerably extend previous research because we considered both women and men and showed that not only mothers but also fathers perceived a decrease of social support across the peripartum period. Thus, it is plausible to assume that both women and men tend to feel overwhelmed by their new parental role after childbirth and thus experience a larger discrepancy between desired and actual levels of support during this challenging time.

Third, we found that more extraverted, emotionally stable, agreeable, conscientious, and open parents felt socially better supported across the peripartum period. As suggested by previous research, higher levels on these traits relate to a range of cognitive, emotional, and interpersonal skills (5, 26, 55), which could ease the transition to parenthood and explain our results. For example, more extraverted parents might have more friends and more agreeable parents more intimate relationships, leading to higher support in the first months postpartum. Emotionally stable mothers and fathers might be particularly resistant against stress, conscientious parents might re-organize their daily routines more effectively, and open individuals might find smart and creative solutions in challenging family situations. Taken together, these mechanisms might lead to a lower need of support (e.g., due to better problem-solving abilities) and higher actual support (e.g., due to closer social relationships) (25).

Fourth, our findings particularly emphasize the importance of extraversion. Extraversion was not only the strongest predictor of perceived social support but also predicted a smaller peripartum decrease of perceived social support. In this regard, it is plausible to assume that more extraverted individuals not only have a larger social network (25). They might also be able to communicate their needs more clearly and actively ask for help from family members, friends, physicians, and childcare professionals (25). In addition, more extraverted mothers and fathers might profit from higher levels of positive affect and social leisure activities in everyday life, which could buffer parenting stress (26).

Finally, our findings were nearly identical for both samples, which underlines the replicability of our results. This fact is particularly noteworthy given the “replication crisis” and increasing claims for more replicable, robust, and reproducible research in psychological science (36). However, the interactive effect with extraversion was found in the DREAM but not in the MARI sample, which might be due to the larger sample size.

### Strengths and Limitations

Our work has several strengths: We used data from two prospective-longitudinal studies in expectant parents. In both studies, the Big Five personality traits were assessed with the BFI, and perceived social support was assessed repeatedly from early pregnancy to late postpartum with the FSozU, a well-established questionnaire. Our findings were highly similar in both samples, which emphasizes the replicability, robustness, and reproducibility of the results.

Nonetheless, the following limitations should be mentioned: First, personality was assessed with the BFI-K (MARI) and BFI-S (DREAM)—short questionnaires that are less comprehensive than longer measures and, for example, do not allow to distinguish between different facets of the Big Five. Second, in DREAM, personality (T2) was assessed after the first assessment of perceived social support (T1), and personality might have changed from T1 to T2. However, the Big Five traits are considered to be relatively stable over short periods of time (56–59). Third, not all parents of the respective baseline sample participated until the last assessment of perceived social support, and systematic drop out might have occurred (38, 39). For example, especially parents with low and decreasing levels of perceived support might have ended their participation earlier, which could have led to an underestimation of peripartum changes in perceived social support (6). Fourth, in both studies, more mothers than fathers participated, and drop out was also lower in mothers vs. fathers. In MARI, participating fathers were approached indirectly *via* participating mothers, which might have doubled selection effects and, for example, might have led to an overrepresentation of particularly engaged fathers (37). Fifth, we focused on peripartum changes in general social support (e.g., from family members, friends, colleagues, or health professionals), considering the role of gender and personality. Additional research could specifically focus on dyadic associations of partner support in participating couples, for instance, based on the actor-partner interdependence model (60). Sixth, our data stem from (expectant) parents in and around Dresden, Germany (39, 40). Thus, the generalizability to mothers and fathers in other regions might be limited.

## Conclusions

Taken together, our findings suggest that especially extraversion plays an important role for high and stable levels of perceived social support across the peripartum period. Therefore, particularly highly introverted mothers and fathers might profit from targeted interventions. Such programs might promote specific skills to establish and maintain social contacts, communicate individual needs, and actively seek for support in stressful situations (7). Future research is needed to investigate the role of extraversion for perceived and actual levels of social support across the peripartum period in greater detail as well as to test the efficacy of targeted interventions. Moreover, future research could examine how romantic relationship characteristics contribute to peripartum changes in partner support and how (expectant) mothers and fathers influence each other reciprocally over time. Finally, the interplay of personality traits with predisposing vulnerabilities in parents at increased risk for mental disorders may be investigated in further detail.

## Data Availability Statement

The data of this study are not openly accessible because after consulting the Ethics Committee and due to the sensitive nature of the questions asked in this study, participants were assured that all raw data would remain confidential and not be shared. Further information on the data can be obtained from the corresponding author, the Ethics Committee of the Medical Faculty of the Technische Universitaet Dresden, and the Institute of Clinical Psychology and Psychotherapy of the Technische Universitaet Dresden. Requests to access the datasets should be directed to julia.martini@tu-dresden.de.

## Ethics Statement

The studies involving human participants were reviewed and approved by the Ethics Committee of the Medical Faculty of the Technische Universitaet Dresden. All participants provided their written informed consent to participate in this study.

## Author Contributions

JM: conception and design of the MARI study and data collection MARI study. SG-N: conception and design of the DREAM study and data collection DREAM study. EA: data analysis and preparation of the manuscript. EA, SG-N, and JM: interpretation, critical review, and approval of the final manuscript. Principle investigator of the DREAM study is SG-N, who is a management committee member of COST action CA18211: DEVOTION: Perinatal Mental Health and Birth-Related Trauma: Maximizing best practice and optimal outcomes. All authors contributed to the article and approved the submitted version.

## Funding

The MARI study was funded by the Institute of Clinical Psychology and Psychotherapy, Technische Universität Dresden, Germany, and supported in parts by the Lundbeck Institute Skodsborg, Denmark. Parts of the field work were additionally supported by the Friends and Sponsors (Gesellschaft der Freunde und Förderer) of the Technische Universität Dresden, Germany. The DREAM study was funded by the German Research Foundation (Deutsche Forschungsgemeinschaft; DFG; Grant Numbers GA 2287/4-1 and GA 2287/4-2). This paper contributes to the EU COST Action 18211 supported by COST (European Cooperation in Science and Technology).

## Conflict of Interest

The authors declare that the research was conducted in the absence of any commercial or financial relationships that could be construed as a potential conflict of interest.

## Publisher's Note

All claims expressed in this article are solely those of the authors and do not necessarily represent those of their affiliated organizations, or those of the publisher, the editors and the reviewers. Any product that may be evaluated in this article, or claim that may be made by its manufacturer, is not guaranteed or endorsed by the publisher.
